# SARS Transmission among Hospital Workers in Hong Kong

**DOI:** 10.3201/eid1002.030534

**Published:** 2004-02

**Authors:** Joseph T.F. Lau, Kitty S. Fung, Tze Wai Wong, Jean H. Kim, Eric Wong, Sydney Chung, Deborah Ho, Louis Y. Chan, S.F. Lui, Augustine Cheng

**Affiliations:** * Chinese University of Hong Kong, Special Administrative Region, People’s Republic of China (SAR); † Hospital Authority, Government of Hong Kong, Hong Kong SAR

**Keywords:** SARS, hospital worker, nosocomial infections, breakthrough transmission, Hong Kong, research

## Abstract

Despite infection control measures, breakthrough transmission of severe acute respiratory syndrome (SARS) occurred for many hospital workers in Hong Kong. We conducted a case-control study of 72 hospital workers with SARS and 144 matched controls. Inconsistent use of goggles, gowns, gloves, and caps was associated with a higher risk for SARS infection (unadjusted odds ratio 2.42 to 20.54, p < 0.05). The likelihood of SARS infection was strongly associated with the amount of personal protection equipment perceived to be inadequate, having <2 hours of infection control training, and not understanding infection control procedures. No significant differences existed between the case and control groups in the proportion of workers who performed high-risk procedures, reported minor protection equipment problems, or had social contact with SARS-infected persons. Perceived inadequacy of personal protection equipment supply, infection control training <2 hours, and inconsistent use of personal protection equipment when in contact with SARS patients were significant independent risk factors for SARS infection.

The first large-scale outbreak of severe acute respiratory syndrome (SARS) occurred on or near March 12, 2003 in the Prince of Wales Hospital in Hong Kong (*1*). In this worldwide epidemic, hospital workers were one of the affected groups; as of May 31, 2003, a total of 384 (22.1%) of 1,739 suspected or confirmed cases reported in Hong Kong were hospital workers (*2*). In the initial phase of the epidemic, hospital workers did not take special protective measures. Thus, hospital workers accounted for 43.6% (68 of 156 cases) of those admitted to the Prince of Wales Hospital from March 11 to 25, 2003 (*3*). By May 25, 2003, a total of 453 confirmed SARS cases had been admitted to hospitals in the New Territories East cluster of the Hospital Authority in Hong Kong, which serves 1.3 million people and to which the Prince of Wales Hospital belongs. From March 28, 2003 to May 29, 2003, a total of 77 cases of SARS infection among hospital workers had been reported by the 5 hospitals in the cluster.

A recent study concluded that the use of protective masks is an effective countermeasure against SARS (*4*). Nevertheless, even after these measures were implemented, there were approximately 300 more hospital workers in whom the disease developed. Limitations of that study were the small number of cases and potential confounding by the possible differences in the intensity of care given to the SARS patients between the case and control groups.

Breakthrough transmission continues despite implementing strict infection control measures. We investigated the factors associated with breakthrough transmission of the SARS virus among hospital workers infected in hospital settings.

## Materials and Methods

### Study Design

A 1:2 matched case-control design was used. All participants were working in wards with SARS inpatients, some of which also included non-SARS patients. The case group included all infected hospital workers in the five hospitals of the New Territories East cluster of the Hospital Authority in Hong Kong who were registered as SARS cases by the Department of Health’s eSARS registry and were hospitalized during March 28 through May 25, 2003.

The SARS case definition criteria used by Hong Kong Hospital Authority is as follows: radiographic evidence of infiltrates consistent with pneumonia, and current fever >38°C or a history of such at any time in the preceding 2 days, and at least two of the following: history of chills in the past 2 days, new or increased cough or breathing difficulty, general malaise or myalgia, typical signs of consolidation, or known exposure. These criteria are equivalent with the World Health Organization’s case definition for probable SARS. Suspected SARS cases are those that do not completely fulfill the above definition but were considered to be likely cases of SARS on the basis of clinical judgment. If no know history of exposure exists, patients are considered for exclusion if an alternative diagnosis can fully explain the clinical symptoms. Laboratory confirmation of SARS infection was also conducted by one or more of the following assays: reverse transcriptase-polymerase chain reaction (RT-PCR); culture from throat wash, urine, stool and nasal swab specimens taken at days 1, 3, and 5; or paired serologic assay from clotted blood taken at day 1 and 21.

Of 77 probable and suspected SARS cases, 72 (93.5%) participated in the study. As all staff was required to use protective masks from March 12, 2003, these hospital workers were presumed to have contracted the virus as a result of breakthrough transmission. An infection control nurse explained the purpose and logistics of the study to the study participants, obtained their verbal consent for participation, presented them with a structured questionnaire, and collected the completed questionnaire. SARS case-patients were asked to nominate as controls two colleagues who had been working in the same job position, in the same ward, and in proximity with the case-patient before he became ill. Medical and nursing staff (48 of 72 cases) self-administered the questionnaires while other staff (e.g., healthcare assistants and ward assistants, etc) were interviewed by an infection control nurse. Out of the 72 cases, 57 nominated 114 controls who completed the questionnaire (114/144 = 79.2%); 15 cases did not nominate a control and hence 30 controls were randomly selected from the duty roster of the day before the case felt unwell, matching for job position (30/144 = 20.8%). Questionnaires were collected from 57 (79.2%) nominated controls. Nominated controls who did not return the questionnaire were replaced by controls randomly selected from the duty roster of the day before the case felt unwell, matching for job position (15/72 = 20.8%). Of the 144 controls completing the questionnaire, one was invalidated because she later became a suspected case. Controls showed neither influenzalike symptoms nor SARS-related symptoms during the study and had not been identified as a suspected SARS case as of August 15, 2003. No blood test was conducted to determine whether these persons were asymptomatic SARS cases. Another study that tested 674 healthcare workers who were working in the same hospital cluster found no asymptomatic or subclinical SARS. It can thereby be assumed that the control group had not contracted the virus (*5*).

### Measurements

Questions were asked about the hospital worker’s job position, whether the healthcare worker had been seconded from another unit, whether he/she had made physical contact with any SARS patients and if so, whether various high-risk procedures were performed to the SARS patient (including intubation, suction, cardiopulmonary resuscitation).

Personal protection equipment use (N95 mask, surgical mask, gloves, goggles, gown, and cap) was examined under three different settings: when having direct contact with SARS patients, when having contact with “patients in general” (includes both SARS and non-SARS patients), and when there was “no patient contact.” Information about the frequency of using different types of personal protection equipment (never, occasionally, most of the time, or all of the time) was asked for each of these three settings. A respondent was considered to be exposed to a particular risk if he or she had “never” or “occasionally” been using personal protection equipment rather than “most or all of the time.” Those who had not been in contact with any SARS patients or patients in general were considered as not having been exposed to the particular risk. Respondents were asked whether they perceived the supply of such personal protection equipment items to be adequate or not (yes/no). Questions regarding the frequency of hand washing after making contact with SARS patients, patients in general and when there was no patient contact (never, occasionally, most of the time, all of the time) were also asked. In the analysis, frequency of using personal protection equipment and frequency of hand hygiene practice were coded into 2 categories: used inconsistently (i.e., “never or occasionally used”) or used consistently (“used most or all of the time”).

Study participants were also asked to assess whether the masks fit them (yes/no), whether their goggles were fogged (yes/no), and the frequency of touching protective masks (never, occasionally, most of the time, or always), and whether they had any problems complying with infection control procedures (yes/no). Respondents were asked whether they had ever made social contact with others who were later found to be SARS cases before SARS-related symptoms manifested (yes/no/not sure), within the 14-day period before the case’s onset of symptoms. The questionnaire also asked about the respondent’s exposure to infection control training (length of SARS infection control training) and whether they understood the infection control measures (yes/no). A trained research assistant contacted the respondents by telephone to follow up on any incomplete or unclear answers.

### Statistical Methods

Unadjusted matched odds ratios calculated from conditional logistic regression methods (*6*) are summarized in Tables 1 to 4. A multivariate conditional logistic regression was fitted using a forward-stepwise procedure with all variables that were marginally significant (p < 0.10) in the unadjusted analyses as candidates for selection. Matched odds ratios and their exact 95% confidence intervals were derived. LogXact for Windows version 4.1 was used for all calculations (*7*).

**Table 1:** Percentage of healthcare workers exposed to the risk of inconsistent use of different types of personal protection equipment in 3 clinical settings with SARS patients^a^

Type of personal protection equipment	Control (n = 143)	%	Case (n = 72)	%	Matched OR (exact 95% CI)	p value (exact)
N95 or Surgical mask^b^						
Direct contact with SARS patient	0	0	1	1.4	2.00 (0.05 to ∞)	0.6667
Direct contact with patients in general ^c^	1	0.7	2	2.8	4.00 (0.21to 235.99)	0.5185
No patient contact^d^	3	2.2	4	5.7	2.43 (0.41 to 16.77)	0.4198
N95^b^						
Direct contact with SARS patients	6	4.2	7	9.7	2.86 (0.70 to 13.71)	0.1683
Direct contact with patients in general^c^	5	3.6	3	4.2	1.28 (0.16 to 10.47)	1.0000
No patient contact^d^	14	10.2	12	17.1	1.83 (0.72 to 4.71)	0.2315
Goggles^b^						
Direct contact with SARS patients	12	8.4	23	31.9	6.41 (2.49 to 19.49)	<0.0001
Direct contact with patients in general^e^	7	5.1	16	22.2	6.93 (2.19 to 28.85)	0.0003
No patient contact^f^	19	13.9	21	30.0	3.50 (1.42 to 9.47)	0.0046
Gown^b^						
Direct contact with SARS patients	6	4.2	15	20.8	8.85 (2.46 to 48.28)	0.0002
Direct contact with patients in general^c^	2	1.4	12	16.7	11.54 (2.56 to 106.36)	0.0002
No patient contact^f^	16	11.7	19	27.1	3.42 (1.38 to 9.30)	0.0061
Gloves^b^						
Direct contact with SARS patients	2	1.4	11	15.3	20.54 (2.96 to 887.72)	0.0002
Direct contact with patients in general^c^	5	3.6	7	9.7	3.53 (0.77 to 21.85)	0.1211
No patient contact^f^	20	14.6	19	27.1	2.42 (1.05 to 5.81)	0.0374
Cap^b^						
Direct contact with SARS patients	8	5.6	17	23.6	7.30 (2.33 to 30.21)	0.0001
Direct contact with patients in general^c^	5	3.6	15	20.8	12.81 (2.92 to 116.75)	0.0001
No patient contact”^f^	16	11.7	22	31.4	4.05 (1.68 to 10.76)	0.0009
No. of equipment inconsistently used with direct contact with SARS patients^g^						
0	129	90.2	45	62.5	1.00	
1–2	7	4.9	13	18.1	5.35 (1.79 to 18.53)	0.0015
>3	7	4.9	14	19.4	7.84 (2.30 to 34.83)	0.0003
No. of equipment inconsistently used with direct contact with patients in general^e, g^						
0	127	92.0	52	72.2	1.00	
1–2	6	4.3	8	11.1	4.85 (1.01 to 31.86)	0.0479
>3	5	3.6	12	16.7	10.83 (2.29 to 102.60)	0.0007
No. of equipment inconsistently used when there was no patient contact ^g, h^						
0	113	82.5	46	65.7	1.00	
1–2	6	4.4	4	5.7	1.56 (0.28 to 7.97)	0.7721
>3	18	13.1	20	28.6	3.40 (1.37 to 9.23)	0.0061

^a^SARS, severe acute respiratory syndrome; CI, confidence interval; OR, odds ratio. ^b^Those having no contact with patients were considered to be unexposed to the tabulated risk factor. ^c^Information on 4 controls missing. ^d^Information on 4 controls and 2 cases missing . ^e^Information on 5 controls missing. ^f^Information on 6 controls and 1 case missing. ^g^Information on 6 controls and 2 cases missing. ^h^Including N95, goggles, gown, gloves and cap.

**Table 4:** Percentage distributions of variables related to training, patient care, social contact and mask compliance^a^

Characteristic	Control (n = 143)		Case (n = 72)		Matched OR (exact 95% CI)	p value (exact)
	n	%	N	%		
Length of SARS infection control training						
None	40	28.0	36	50.0	1.00	
<2hrs	67	46.9	33	45.8	0.47 (0.18 to 1.14)	0.1028
>2hrs	36	25.2	3	4.2	0.03 (0.001 to 0.20)	<0.0001
Understood infection control measures^b^						
Yes	130	91.5	54	76.1	1.00	
No	12	8.5	17	23.9	3.14 (1.35 to 7.73)	0.0065
Acquired updated information						
No	5	3.5	8	11.1	1.00	
Yes	136	96.5	64	88.9	0.27 (0.06 to 1.04)	0.0574
High risk procedures with SARS patients^c^						
No	115	86.5	60	83.3	1.00	
Yes	18	13.5	12	16.7	1.22 (0.45 to 3.14)	0.8061
Direct contact with SARS patients						
No/Not sure	38	26.6	27	37.5	1.00	
Yes	105	73.4	45	62.5	0.57 (0.28 to 1.14)	0.1197
Direct contact with patients in general						
No/Not sure	7	4.9	3	4.2	1.68	1.000
Yes	136	95.1	69	95.8	(0.07 to 117.74)	
Seconded from another unit						
No	77	53.8	46	63.9	1.00	
Yes	66	46.2	26	36.1	0.60 (0.29 to 1.21)	0.1671
Social contact with SARS patients						
No/Not sure	95	66.4	55	76.4	1.00	
Yes	48	33.6	17	23.6	0.59 (0.28 to 1.19)	0.1592
Frequency of touching the N95^d^						
Most of the time/Always	33	23.4	19	29.2	1.32 (0.63 to 2.74)	0.5205
General problems with mask^e^	68	48.6	28	40.6	0.66 (0.34 to 1.27)	0.2407
Problems with mask fit^f^	70	49.0	33	47.8	1.00 (0.51 to 1.95)	1.0000
Problems with fogging of goggles^g^	75	52.8	26	40.0	0.61 (0.31 to 1.17)	0.1520
Overall problems in general compliance^h^	69	50.0	29	41.4	0.58 (0.25 to 1.33)	0.2264

^a^OR, odds ratio; CI, confidence interval; SARS, severe acute respiratory syndrome. ^b^Information on 1 control and 1 case missing. ^c^Information on 10 controls with direct contact with SARS patients missing. ^d^Excluded 2 controls and 6 cases who did not use N95 mask; information on 1 case missing. ^e^Excluded 1 case who did not use mask; information on 3 controls and 2 cases missing. ^f^Excluded 1 case who did not use mask; information on 2 cases missing. ^g^Excluded 3 cases who did not use goggle; information on 1 control and 3 cases missing ^h^Excluded 1 case who did not use any equipment; information on 5 controls and 1 case missing

## Results

### Background Characteristics of Respondents

The 72 SARS-infected healthcare workers worked in five hospitals (distribution: 50% Alice Ho Miu Ling Nethersole Hospital, 40.3% from Prince of Wales Hospital, 2.8% from North District Hospital, 4.2% from Shatin Hospital, and 2.8% from Taipo Hospital). The study sample was composed of nurses 59.7% (n = 43), healthcare assistants 23.6% (n = 17), medical officers 9.7% (n = 8), clerical staff (2.8%, n = 2), and workmen (4.2%, n = 3).

### Use of Masks and Other Types of Protection Equipment

Almost 100% of the study respondents used either an N95 mask or surgical mask in all 3 settings (Table 1). The differences of the use of the N95 mask (most of those not wearing a N95 mask were wearing a surgical mask) were not statistically significant between cases and controls in any of the three settings (p > 0.05, Table 1).

When hospital workers were in direct contact with SARS patients, the case group was more likely to inconsistently use goggles (odds ratio [OR] = 6.41, p < 0.0001), gowns (OR = 8.85, p = 0.0002), gloves (OR = 20.54, p = 0.0002), and caps (OR = 7.30, p = 0.0001) than the control group. When in direct contact with patients in general, cases were more likely to inconsistently use goggle (OR = 6.93, p = 0.0003), gowns (OR = 11.54, p = 0.0002), and caps (OR = 12.81, p = 0.0001). When there was “no patient contact,” cases had more than a twofold likelihood of inconsistently using goggles (p = 0.0046), gowns (p = 0.0061), gloves (p = 0.0374), or cap (p = 0.0009), compared to their matched controls. Having three or more personal protection equipment inconsistently used (including masks) was also a significant predictor of SARS infection for hospital workers in direct contact with SARS patients (OR = 7.84, p = 0.003); for those with direct contact with patients in general (OR = 10.83, p = 0.0007); and for those with no patient contact (OR = 3.4, p = 0.006) (Table1).

More than 97% of both the cases and control group consistently reported to practice good hand hygiene after contacting SARS patients or “patients in general” therefore differences between the two groups were not statistically significant (p = 0.22, and p = 1.00, respectively, Table 2). There was, however, a statistically significant difference in the proportion of cases (14.3%) and controls (2.1%) of hospital workers who reported inconsistent hand hygiene when there was “no patients contact” (OR = 6.38, 95% CI = 1.64, 36.2, p = 0.0044).

**Table 2:** Percentage with inconsistent hand hygiene^a^

Category	Control (n = 143)		Case (n = 72)		Matched OR (exact 95% CI)	p value (exact)
	n	%	n	%		
After direct contact with SARS patients	0	0	2	2.8	4.83 (0.38 to ∞)	0.2222
After direct contact with “patients in general”^b^	2	1.4	1	1.4	1.00 (0.02 to 19.21)	1.0000
When there was “no patient contact”^c^	3	2.1	10	14.3	6.38 (1.64 to 36.17)	0.0044

^a^OR, odds ratio; CI, confidence interval; SARS, severe acute respiratory syndrome. ^b^Information on 3 controls missing**.** ^c^Information on 1 control and 2 case missing.

### Perceived Inadequacy of Personal Protection Equipment Supply

A much higher percentage of SARS cases compared to controls reported a perceived inadequate supply of each of the 6 types of personal protection equipment (OR = 28.0, p < 0.0001 for surgical masks; OR = 5.19, p = 0.0004 for N95 masks; OR = 8.44, p < 0.0001 for gowns; OR = 29.3, p < 0.0001 for gloves; OR = 19.8, p < 0.0001 for goggles; OR = 52.4, p < 0.0001 for cap) (Table 3). Most notably, 44.4% of the cases reported that there was an inadequate supply of at least one item of the personal protection equipment, as compared to 14.0% of the controls (OR = 6.78, p < 0.0011); among SARS cases, 26% reported three or more personal protection equipment items as being in inadequate supply, compared to 1.4% of the controls (OR = 52.2, p < 0.0001).

**Table 3:** Percentages with perceived inadequacy of personal protection equipment supply and breakthrough SARS infection among hospital workers^a^

Type of personal protection equipment	Control (n = 143)		Case (n = 72)		Matched OR (exact 95% CI)	p value (exact)
	n	%	n	%		
Surgical mask	1	0.7	14	19.4	28.00 (4.26 to ∞)	<0.0001
N95 mask	13	9.1	20	27.8	5.19 (1.95 to 16.13)	0.0004
Gown	7	4.9	19	26.4	8.44 (2.77 to 34.37)	<0.0001
Gloves	2	1.4	12	16.7	29.34 (4.79 to ∞)	<0.0001
Goggles	5	3.5	22	30.6	19.81 (4.83 to 174.55)	<0.0001
Cap	4	2.8	21	29.2	52.41 (9.08 to ∞)	<0.0001
Any one of above as inadequate^b^	20	14.0	32	44.4	6.78 (2.86 to 18.51)	<0.0001
No. of items identified to be inadequate^b^						
0	123	86.0	40	55.6	1.00	
1–2	18	12.6	13	18.1	3.25 (1.17 to 9.80)	0.0209
3	2	1.4	19	26.4	52.24 (7.70 to 2280.07)	<0.0001

^a^SARS, severe acute respiratory syndrome; OR, odds ratio; CI, confidence interval. ^b^Including N95 mask, goggle, gown, gloves and cap

### SARS-Related Infection Control Training

The unadjusted results indicated that 50% of SARS cases did not receive any SARS infection control training (versus 28% of the controls) (Table 4). Those who underwent >2 hours of training (4.2% of cases and 25.2% of controls) were far less likely to have been infected with SARS (OR = 0.03, p < 0.0001). Of the SARS cases, 23.9% indicated that they did not understand the infection control measures, compared with 8.5% of the controls (OR = 3.14, p = 0.0065). Duration of SARS training (<2 hrs versus >2 hours) was significantly associated with reported understanding of the infection control measures (OR = 7.29, p = 0.001). There was also a marginal statistically significant difference (OR = 0.27, p = 0.057) in the proportion who reported having received updated SARS information between cases (88.9%) and controls (96.5%).

### Patient Care and Infection Control Measures

A higher but statistically nonsignificant percentage of the control group (73.4%) reported having direct contact with SARS patients as compared to the case group (62.5%). Three (4.2%) of 72 cases and 7 (4.9%) of 143 controls reported that they had no direct contact with patients in general (p > 0.05). Having performed high-risk procedures on SARS patients and being seconded from another unit were not significantly associated with risk of SARS infection (Table 4).

There were no significant differences between the percentages of cases and controls who reported the following problems: general compliance problems, frequency of touching or adjusting the N95 mask, general problems with mask, problems with mask fit, and problems with fogging of goggles (Table 4).

### Social Contact with SARS Cases

Approximately 23.6% of the SARS cases and 33.6% of the matched controls reported ever having social contact with someone who was later diagnosed with SARS before the onset of symptoms of the relevant case (p = 0.1592) (see Table 4).

### Problems Encountered

Seven problems in the unadjusted analysis (Table 5) were significantly associated with risk for SARS infection. An indicator variable was constructed by counting the number of problems encountered by the study participants. Almost all (98.6%) of the case group encountered at least one problem (versus 79.9% in the control group). The risk increases greatly with the number of problems encountered (OR = 44.2 for 3 or more problems, p < 0.0001) (Table 5). Using a cut-off point of two or more problems to predict SARS infection gives a sensitivity and specificity of 0.681 and 0.691, respectively.

**Table 5:** Percentage distribution of the number of problems encountered by the hospital worker^a^

Number of problems encountered^b^	Control			Case			Matched OR (exact 95% CI)	p value (exact)
	n	%	Cumulative %	n	%	cumulative %		
0	27	20.1	20.1	1	1.4	1.4	1.00	
1	65	48.5	68.6	21	30.4	31.8	8.47(1.37 to ∞)	0.0169
2	24	17.9	86.5	17	24.6	56.4	17.78(2.67 to ∞)	0.0010
>3^c.d^	18	13.4	100.0	30	43.5	100.0	44.15(7.02 to ∞))	<0.0001

^a^Excluded nine controls and thee cases that had at least one missing entry on one of the problems encountered. ^b^The seven problems are: 1) inconsistent use of at least 1 type of personal protection equipment when having contact with SARS patients, 2) with “patients in general,” 3) when there was “no patient contact,” 4) when SARS infection control training was less than 2 hours, 5) when the respondent reported not understanding SARS infection control procedures, 6) when at least one personal protection equipment was perceived to be in inadequate supply in the 3 settings, and 7) when hand hygiene was inconsistent when there was “no patient contact.” ^c^Percentages of the number of problems encountered in the control group: 3 problems (6.7%), 4 problems (4.5%), 5 (1.5%), 6 (0.7%), and 7 (0%). ^d^Percentages of the number of problems encountered in the case group: 3 problems (10.1%), 4 (8.7%), 5 (13.0%), 6 (8.7%), and 7 (2.9%).

### Multivariate Analysis

The results of the forward stepwise conditional logistic regression model using the seven significant variables as candidate variables indicate that the perceived inadequacy of personal protection equipment supply (adjusted OR = 4.27, 95% CI 1.66 to 12.54, p = 0.0028), SARS infection control training <2 hours or no training (adjusted OR = 13.6, 95% CI 1.24 to 27.50, p = 0.002), and inconsistent use of more than one type of personal protection equipment when having direct contact with SARS patients (adjusted OR = 5.06, 95% CI 1.91 to 598.92, p=0.02) were significantly and independently associated with SARS infection among hospital workers.

## Discussion

Breakthrough transmission was likely responsible for the SARS infection of these cases, as protective masks (primarily N95) were used consistently by almost all of the cases. All workers were required to wear protective masks from March 12, 2003. Using protective masks alone is, therefore, not sufficient to eliminate SARS transmission among hospital workers. Cases were less likely to have had direct contact with a SARS patient than controls, suggesting that direct physical contact with SARS patients was not necessary for breakthrough transmission to occur. It also suggests that modes of transmission other than droplets cannot be excluded. Consistent hand hygiene after contact with patients was almost universal and was not a significant factor predicting SARS transmission in our study, although hand hygiene appeared to be a risk factor in situations when there was no patient contact.

Data from all the three settings show that inconsistent use of gown, cap, and goggles were all very strongly associated with breakthrough transmissions. Personal protection equipment should be used consistently in all 3 settings. The high degree of collinearity in the use of the various types of personal protection equipment makes it difficult to ascertain which type of personal protection equipment is most important as a SARS countermeasure. Nevertheless, policy makers should be made aware that the supply of different types of personal protection equipment had often been seen as inadequate, and it is one of the very significant risk factors identified. The perception of inadequate supply was not verified by this study. These perceptions may reflect the actual situation or may be an inaccurate impression of the hospital workers. Caution is advised in interpreting these results. Nevertheless, at the time of the study, the media had reported frequent complaints about personal protection equipment supply shortages from hospital workers. The perception of inadequate personal protection equipment is likely to be associated with the personal protection equipment supply situation. Given the large differences in our results (OR > 5.0, p < 0.001), it is likely that personal protection equipment shortages were at least partially responsible for many of the SARS infections. As inadequate knowledge of SARS infection control (“did not understand procedures”) is also a strong risk factor for breakthrough transmission, SARS infection control training must not be overlooked. In-depth, thorough training (>2 hrs) is required.

Soon after the initial SARS outbreak, it was mandatory for all hospital workers to attend at least one 1-hour structured training session delivered by the infection control team, and the records of these sessions were collected and submitted to the Hospital Authority. These training sessions were conducted twice per day for the initial week from the middle of March and on daily basis up to the end of June. The content of these training sessions included basic knowledge of SARS and its clinical presentation, route of transmission, types and proper use of different personal protective equipment for different risk levels, the procedures for handling high risk specimens, environmental disinfection protocols, and commonly observed problems. The content of the training was regularly revised with updated information. Regular updates and attendance of the training sessions were strongly recommended. The unit supervisors were given more intensive training to train their staff. The findings of this study underscore the importance of in-depth training in SARS prevention among hospital workers.

The findings eliminate a number of speculated risk factors which include the following: performing particular high-risk procedures on SARS patients, having social contacts with people who were later found to be SARS cases, and experiencing various minor problems in using the mask. Performing high-risk procedures was not a significant factor, hence, it is speculated that this is due to a high degree of awareness and caution taken when performing these procedures with SARS patients.

It is found that those who encountered any of the 7 identified problems had a greatly increased likelihood of contracting SARS. The number of problems encountered is a good predictor of SARS infection. It is recommended to have health workers complete a checklist of these items after each day’s work, and the management should review these forms. No hospital staff should be exposed to SARS before receiving adequate training or before they have obtained a thorough understanding of the infection control procedures. The results of the multivariate analysis show that infection control training, personal protection equipment use, and perceived supply were independently associated with SARS infection risk among hospital workers.

This study has a number of limitations. As a case-control study, it is subject to recall bias. However, the recall period was usually within 1 week as all the cases were interviewed while they were hospitalized. Hand hygiene data were self-reported and not audited. Nevertheless, since respondents were required to report the frequency of hand washing from a categorical response format rather than an open ended question, the responses should be reliable. Another possible bias may be the case group’s attributing their infection to external factors (e.g., inadequate supplies) and the control group’s doing the opposite. Given that the odds ratios obtained were strongly significant and consistent with one another, it is unlikely that this form of bias could account for all of the observed differences. The study, however, has a relatively large sample size, a high response rate, and has controlled for the exposure to other background confounding factors.
